# A gradient field defeats the inherent repulsion between magnetic nanorods

**DOI:** 10.1098/rsos.140271

**Published:** 2014-10-08

**Authors:** Yu Gu, Ruslan Burtovyy, John Custer, Igor Luzinov, Konstantin G. Kornev

**Affiliations:** Department of Materials Science and Engineering, Clemson University, Clemson, SC 29634, USA

**Keywords:** magnetic nanorods, field gradient, assembly, dynamic systems

## Abstract

When controlling the assembly of magnetic nanorods and chains of magnetic nanoparticles, it is extremely challenging to bring them together side by side while keeping a desired spacing between their axes. We show that this challenge can be successfully resolved by using a non-uniform magnetic field that defeats an inherent repulsion between nanorods. Nickel nanorods were suspended in a viscous film and a non-uniform field was used to control their placement. The in-plane movement of nanorods was tracked with a high-speed camera and a detailed image analysis was conducted to quantitatively characterize the behaviour of the nanorods. The analysis focused on the behaviour of a pair of neighbour nanorods, and a corresponding dynamic model was formulated and investigated. The complex two-dimensional dynamics of a nanorod pair was analysed analytically and numerically, and a phase portrait was constructed. Using this phase portrait, we classified the nanorod behaviour and revealed the experimental conditions in which nanorods could be placed side by side. Dependence of the distance between a pair of neighbour nanorods on physical parameters was analysed. With the aid of the proposed theory, one can build different lattices and control their spacing by applying different field gradients.

## 2. Introduction

In the past decade, one-dimensional magnetic nanostructures, such as magnetic nanorods, chains of magnetic nanoparticles and nanotubes filled with magnetic nanoparticles, have offered great opportunities for the design of multi-functional devices and for the manufacturing of anisotropic nano- and microstructures [1–4]. These applications include, for example, optofluidics [5–9], microrheology [10–14], magnetic swimming [15–20], photonics [21], drug delivery [22] and electromagnetic shielding [23]. Particularly, in the manufacturing of composite materials, different configurations of magnetic fields are usually applied to obtain the desired pattern of magnetic rods or chains [2,4,24–27]. A uniform magnetic field is usually used to align the nanorods in one direction or to form self-assembled chains from magnetic nanoparticles. Recently, the strategies for aligning an assembly of non-interacting magnetic nanorods in both Newtonian and non-Newtonian fluids under a uniform magnetic field have been proposed and developed [28,29]. However, in many cases, one needs to deal with a concentrated colloid of magnetic nanorods where the interactions between nanorods are crucial for the patterning of the microstructures [30–36].

The main challenge when attempting to control the assembly of magnetic nanorods is bringing them together and placing them side by side [7,35,36]. Indeed, when two identical magnetic nanorods come together side by side, they are prone to move away owing to their inherent repulsion. A uniform magnetic field keeps them parallel to each other but they tend to form a tandem with a head-to-tail ordering. Phase diagrams for the long nanorods demonstrate a significant enlargement of the region of repulsion compared with the point dipoles [7,37]. Therefore, one needs to develop a new strategy in order to defeat this inherent repulsion.

**Figure 1.**
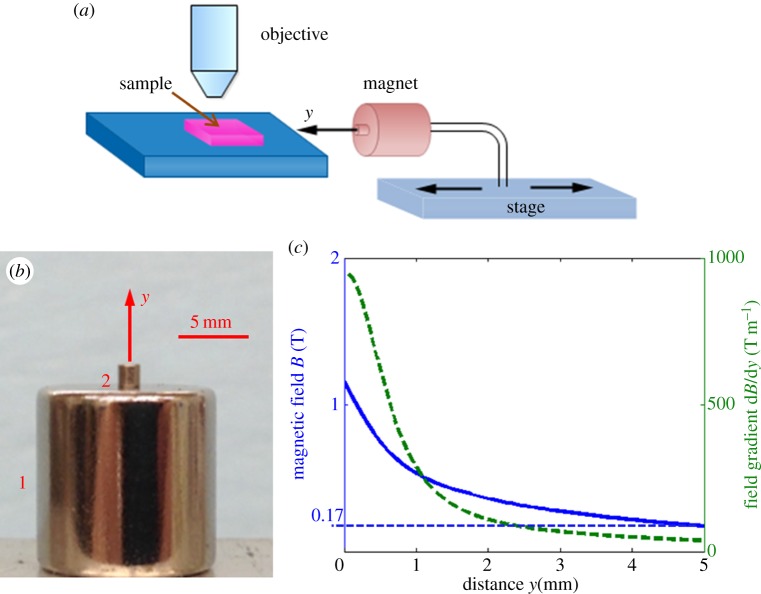
(*a*) Schematic of the experimental set-up. A sample with the nanorod dispersion is placed under the objective and the magnetic field is varied by moving the stage with the attached magnets along the *y*-axis back and forth. (*b*) The system of two magnets used in experiments. (*c*) The magnetic field and its gradient calculated along the *y*-axis of the system. Solid blue line represents magnetic field and dashed green line, field gradient.

One possible strategy is to use a non-uniform magnetic field with a field gradient [30,33,35,38]. In a non-uniform magnetic field generated by a magnet, the magnetic force acting on a nanorod with magnetization vector ***M*** is written as ***F***=*V* (***M***⋅∇)***B***, where ***B*** is the magnetic field vector and *V* is the volume of the nanorod. As follows from this formula, magnetic nanorods can be pushed towards each other by generating a special field gradient. For example, in the region close to the axis of a cylindrical magnet, the radial component of the magnetic field *B*_*r*_ is much weaker than the axial component *B*_*y*_, *B*_*r*_≪*B*_*y*_, where the *y*-axis is taken along the axis of the cylinder. The magnetic force can be approximately calculated as ***F***=*V* (*M*∂/∂*y*)***B***, where *M* is the magnetization of the nanorod. For a cylindrical magnet like the one shown in figure 1*a,b*, the radial component of the magnetic field is positive (*B*_*r*_>0) and its gradient is negative along the *y*-axis (d*B*_*r*_/d*y*<0). Thus, the radial component of magnetic force *F*_*r*_ is negative (*F*_*r*_<0) and the nanorods tend to cluster at the central axis. These arguments show that the placement of magnetic nanorods next to each other can be accomplished by applying a non-uniform magnetic field with a strong axial gradient of the radial component of a magnetic field [39].

However, such strong gradients are difficult to produce at the micrometre scale. On the other hand, the gradient d*B*_*y*_/d*y* of the axial component of a magnetic field is much easier to create and control. Taking advantage of this type of field non-uniformity, various microstructures have already been successfully fabricated [30,33,35], and interest in using this type of field gradient is growing [3,9,30,33,34,40–42]. However, owing to the lack of complete understanding about the behaviour of nanorods in a strong d*B*_*y*_/d*y* gradient, the inter-rod spacing is still difficult to control [2,9,43].

In this paper, we overcome this challenge by modelling the behaviour of a pair of magnetic nanorods in a non-uniform magnetic field with a d*B*_*y*_/d*y* gradient. We analyse the dynamics of the nanorods suspended in a two-dimensional Newtonian film. The nanorods are pulled together by the d*B*_*y*_/d*y* gradient. They also interact with each other through their magnetic poles. In experiments, we use nickel nanorods; their dynamics is filmed using dark field microscopy. We develop a particle-tracking algorithm to follow the nanorod movement and analyse the nanorod trajectories. The analysis of experiments shows that the model adequately describes the behaviour of interacting magnetic nanorods. The conditions for controlled placement of magnetic nanorods were theoretically studied and experimentally examined.

## 3. Experiments

### 3.1 Preparation of a dispersion of nickel nanorods

To make nickel nanorods, we used electrochemical template synthesis [44,45]. Nanorods were synthesized inside 200 nm pores of alumina membranes (Watman Ltd.). We exactly followed the procedure described in the supporting information of Tokarev *et al.* [11]. This experimental protocol enables one to produce nanorods of approximately 6 μm in length and less than 200 nm in diameter. Following the protocol as described in [28,40], we stabilized the nanorods with a layer of polyvinylpyrrolidone (PVP). The PVP-coated nickel nanorods were dispersed in a 76 wt% water–glycerol mixture (76% glycerol, 24% water). In experiments, a dispersion of nickel nanorods of low concentration (0.005 wt%) was used. A 1 μl droplet of this dispersion was placed on a glass slide (VWR International, LLC) and immediately covered by another glass cover slide (VWR International, LLC). Two 26 μm thick Nylon fibres were placed as spacers between slides. This gap thickness was chosen in order that the effect of hydrodynamic interactions between the nanorods and the substrates could be neglected. In our earlier publication [28], we confirmed that the chosen gap thickness is optimal; measuring viscosity of different standard liquids with same set-up and using magnetic rotational spectroscopy, we reproduced viscosity of these standards [11,46].

### 3.2 Optical cell

The schematic of the optical cell is shown in figure 1*a*. Two cylindrical magnets were used in this experiment. The rear face of the smaller magnet was attached to the front face of the larger one so that both magnets had a common axis as shown in figure 1*b*. The smaller magnet was 1.6 mm in diameter and 1.6 mm in length, and the larger magnet was 12.7 mm in diameter and 12.7 mm in length (Grade N52, K&J Magnetics). This construction allowed for a sufficiently strong field of the order of 1 T. The gradient changes by as much as two orders of magnitude within a distance of approximately 5 mm from the front face of the smaller magnet as illustrated in figure 1*c*. The field distribution was simulated using COMSOL, taking magnetization as 1.48×10^6^ A m^−1^ for both magnets and placing the origin of coordinates at the free surface of the small magnet 2.

This construct was positioned under the microscope (Olympus BX 51) with the common axis parallel to the optical stage. The position of the construct was controlled by a linear stage (VT-21, MICOS). The Olympus BX 51 microscope was equipped with a digital camera (SPOT Imaging Solutions, Inc.) allowing for the application of dark field imaging. The sample was positioned under the microscope in front of the smaller cylindrical magnet as shown in figure 1*a*, and the behaviour of nanorods was studied by focusing the camera on four different spots along the common axis of the magnets at the following positions: *y*=5, 3, 2 and 1.5 mm.

### 3.3 Experimental protocol

**Figure 2.**
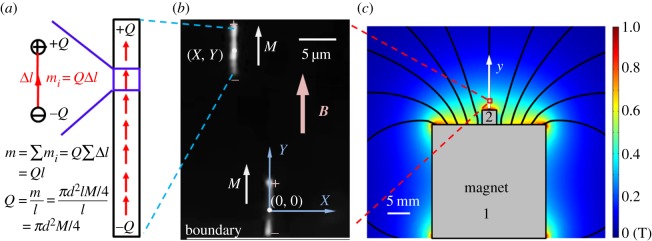
(*a*) Illustration of the model of ‘magnetic charges’ applied to a nanorod with magnetization *M*, length *l* and diameter *d*. The nanorod can be subdivided onto a system of elementary cylinders of lengths Δ*l* each carrying magnetization *m*_*i*_. Only end cylinders have non-compensated ‘charges’ *Q* generated at their faces. (*b*) Illustration of the system of two nanorods. The centre of local Cartesian system of coordinates (*X*, *Y*) is attached to the centre of mass of the nanorod that is pinned to the boundary shown at the bottom of this picture. The position of the incoming nanorod is identified by the coordinates of its centre mass (*X*, *Y*). (*c*) Distribution of the field lines generated by two magnets 1 and 2. Different colours are used to distinguish the strength of magnetic field at different places. The red box marks the position of the spot where figure (*b*) was taken.

The main challenge in studying the interactions between nanorods subject to a non-uniform field is that the nanorods always move within the fluid. The nanorods keep moving towards the region of a stronger field until they reach a boundary, e.g. the liquid–air interface. When a nanorod hits a boundary, it does not move anymore and stays pinned to the boundary. It is therefore convenient to focus the camera to a pinned nanorod and watch the behaviour of incoming nanorods (figure 2*b*).

**Figure 3.**
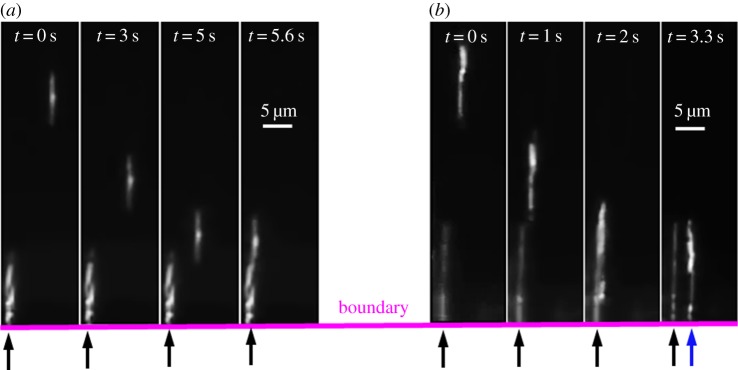
Two series of images illustrating different scenarios of the nanorod landing. (*a*) The incoming nanorod lands on top of the pinned one. (*b*) The incoming nanorod lands side by side next to the pinned one.

Initially, the magnets were placed far away from the sample to eliminate any translational motion of the nanorods. The recording started when the magnets were brought closer to the sample. Figure 3 shows two sequences of images illustrating different scenarios of the nanorods landing: (*a*) landing on top of the pinned nanord and (*b*) landing next to each other.

## 4. Magnetostatic interactions between nanorods and external field

In the focal plane of observation, it is convenient to introduce the local Cartesian system of coordinates (*X*, *Y*) with the *Y*-axis aligned along the common axis of the magnets. The origin of the local system of coordinates is taken at the centre of mass of the pinned nanorod as illustrated in figure 2*b*. The position of the incoming nanorod is measured by its centre of mass (*X*, *Y*). The length of the pinned nanorod and the length of the incoming nanorod are denoted as *L* and *l*, respectively.

As illustrated in figure 2*c*, the *x*-component of the magnetic field ***B*** is almost zero at these points. Therefore, the magnetization ***M*** of all nanorods is expected to point in the *y*-direction. If the nanorods were non-interacting, they would move only in the *y*-direction. Within the small field of view (approx. 20×20 μm^2^) shown in figure 2*b*, the d*B*_*y*_/d*y* gradient is almost constant, d*B*_*y*_/d*y*=*α*. For a strong gradient (*α*∼100 T m^−1^), the variation of the field over a 20 μm distance is approximately *B*_*y*_∼2 mT. On the other hand, the magnitude of the field is greater than |*B*|>0.1 T. Therefore, we assume that the magnetization of nanorods is constant and that the two neighbour nanorods have the same magnetization ***M***. In experiments, the applied magnetic field was sufficiently strong to ensure that the nanorods were not able to change their orientations even in close proximity to each other.

As the distance between the nanorods is comparable with their lengths, the nanorods cannot be treated as point dipoles [7]. We therefore use the model of ‘magnetic charges’ [7,47,48]. To determine the charge *Q*, we divide the magnetic nanorod into a chain of infinitesimally small magnets as shown in figure 2*a*. Each elementary magnet has the moment *m*_*i*_=*QΔl* with an elementary ‘magnetic charge’ *Q*. If we sum up all elementary magnets within the nanorod, all internal poles of the opposite sign will be cancelled out and ‘magnetic charges’ of the opposite sign will remain only at the ends of the nanorod. For a nanorod with diameter *d* and magnetization *M*, the ‘magnetic charge’ *Q* can be therefore calculated as: *Q*=*πd*^2^*M*/4.

Following the chosen system of coordinates, figure 2*b*, the energy of the nanorod subject to an external field can be calculated by introducing magnetostatic potential *φ*(*X*, *Y*). It has to satisfy the Laplace equation written in cylindrical coordinates as: ∂^2^*φ*/∂*X*^2^+(1/*X*)(∂*φ*/∂*X*)+∂^2^*φ*/∂*Y* ^2^=0. In the vicinity of the central axis, the potential is represented as: *φ*=−*B*_0_*Y* −*α*(*X*^2^−2*Y* ^2^)/4, where *B*_0_ is the constant component of a non-uniform external magnetic field taken at the centre of mass of the pinned nanorod (0, 0). In our experiments, the gradient *α*=−d*B*_*y*_/d*y* is always positive. This implies that the nanorods tend to move to the boundary (*X*, −*L*/2) as illustrated in figure 2*b*.

With the given potential, the magnetic field is obtained as ***B***=−∇*φ*=(*αX*/2,*B*_0_−*αY*). The magnetostatic energy of the incoming nanorod in the external magnetic field is calculated as: *φ*(*X*,*Y* +*l*/2)*Q*−*φ*(*X*,*Y* −*l*/2)*Q*=*QlY* *α*−*QlB*_0_. The second term is independent of the position of the incoming nanorod, and hence it does not contribute to the force balance. The total magnetostatic energy of two interacting magnetic nanorods under the field gradient is therefore written as:

4.1 $$\begin{array}{rl} U(X,Y) & =\frac{\mu_{0}Q^{2}}{4\pi }(\frac{1}{\sqrt{X^{2}+{\left(Y+l/2-L/2\right)}^{2}}}-\frac{1}{\sqrt{X^{2}+{\left(Y-l/2-L/2\right)}^{2}}}\\ & \;-\frac{1}{\sqrt{X^{2}+{\left(Y+l/2+L/2\right)}^{2}}}+\frac{1}{\sqrt{X^{2}+{\left(Y-l/2+L/2\right)}^{2}}})+QlY\alpha , \end{array}$$

where *μ*_0_ is the permeability of a vacuum. As follows from equation (3.1), magnetostatic energy scales as *U*∝*μ*_0_*Q*^2^/(4*πL*). We can make equation (3.1) dimensionless by dividing it by *μ*_0_*Q*^2^/(4*πL*), provided that all coordinates (*X*, *Y*) are normalized by the length of the pinned nanorod *L*:

4.2 $$\begin{array}{rl} \frac{4\pi LU(X,Y)}{\mu_{0}Q^{2}} & =(\frac{1}{\sqrt{{\left(X/L\right)}^{2}+{\left(Y/L+(l/2L)-1/2\right)}^{2}}}-\frac{1}{\sqrt{{\left(X/L\right)}^{2}+{\left(Y/L-(l/2L)-1/2\right)}^{2}}}\\ & \;-\frac{1}{\sqrt{{\left(X/L\right)}^{2}+{\left(Y/L+(l/2L)+1/2\right)}^{2}}}+\frac{1}{\sqrt{{\left(X/L\right)}^{2}+{\left(Y/L-(l/2L)+1/2\right)}^{2}}})+\beta \frac{Y}{L}. \end{array}$$

The factor *β*=4*πL*^2^*α*/(*μ*_0_*Q*) measures the strength of the field gradient with respect to the energy of mutual magnetostatic interactions between two nanorods. When this parameter is small, *β*≪1, the field gradient has almost no effect on the incoming nanorod and the two nanorods interact as if there was no field gradient. If this parameter is large, *β*≫1, the incoming nanorod should not feel any presence of the neighbour nanorod hence it should be able to land on the boundary.

**Figure 4.**
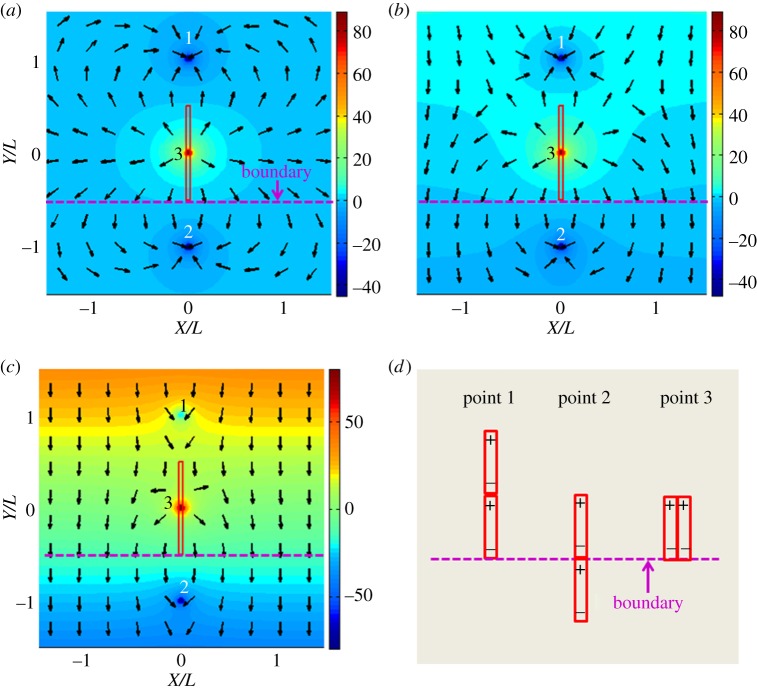
The energy landscape for an incoming nanorod positioned at (*X*, *Y*). The pinned nanorod has the same magnetization, length and diameter. (*a*) The energy landscape for a nanorod subject to the field generated by the pinned nanorod when the external field is not applied, *β*=0. (*b*) Deformation of the energy landscape caused by the field gradient corresponding to *β*=2.5. (*c*) Effect of a strong gradient *β*=25. Different colours represent different energy levels. The black arrows show the direction of magnetic force acting on the incoming nanorod. The dashed purple line shows the impermeable boundary. (*d*) Configurations 1 and 2 correspond to the energy minima, and configuration 3 corresponds to the energy maximum when the nanorods interact in the absence of external field.

Figure 4 shows the energy landscape generated by the incoming nanorod in the presence of the pinned nanorod and external magnetic field ***B***=(*αX*/2,*B*_0_−*αY*). In calculations, we assumed that the two nanorods have the same length, *L*=*l*. Different colours correspond to the different energy levels.

Using this energy landscape, one can calculate the force acting on the incoming nanorod. The magnetic force is obtained through the gradient of the total magnetostatic energy *U*(*X*, *Y*) as

4.3 $$F_{X}=\frac{-\partial U(X,Y)}{\partial X}\;\text{and}\;F_{Y}=\frac{-\partial U(X,Y)}{\partial Y}.$$

The black arrows in figure 4 show the direction of magnetic force acting on the incoming nanorod.

In the absence of an external magnetic field gradient, *β*=0, the nanorods tend to come together forming a head-to-tail configuration (figure 4*a*). This case corresponds to points 1 (0, 1) and 2 (0, −1) in figure 4*d* providing the same energy level. Owing to the impermeable boundary, the incoming nanorod cannot reach point 2. Therefore, point 1 is energetically favourable because two opposite ‘magnetic charges’ cancel each other at the junction point.

In the absence of a magnetic field gradient, when two nanorods are placed side by side, point 3 (0, 0) in figure 4*d*, they produce the energy maximum. Strong repulsion between the nearest ‘magnetic charges’ of the same sign forces the nanorods to run away from this configuration 3.

In a field gradient, the energy landscape deforms, and one finds new minima. These minima appear as a result of topological transformations of the energy surface as illustrated in figure 4*b,c*. When the field gradient is not strong and parameter *β* is of the order of 1, the deformation of the energy surface is insignificant, yet the topography changes to decrease the region with a strong *x*-component of the magnetic force. This change occurs in regions |*X*/*L*|>1, where the magnetic force undergoes significant change. The arrows in figure 4*b*, *β*=2.5, show that the *x*-component of the force becomes small in these regions. Therefore, the incoming nanorod is expected to land at the boundary and the static friction offered by the boundary is sufficient to keep nanorods from moving in the *x*-direction. Points 1 and 2 are still the energy minimum and point 3 is the energy maximum. The field gradient causes the energy level at point 2 to decrease relative to point 1.

**Figure 5.**
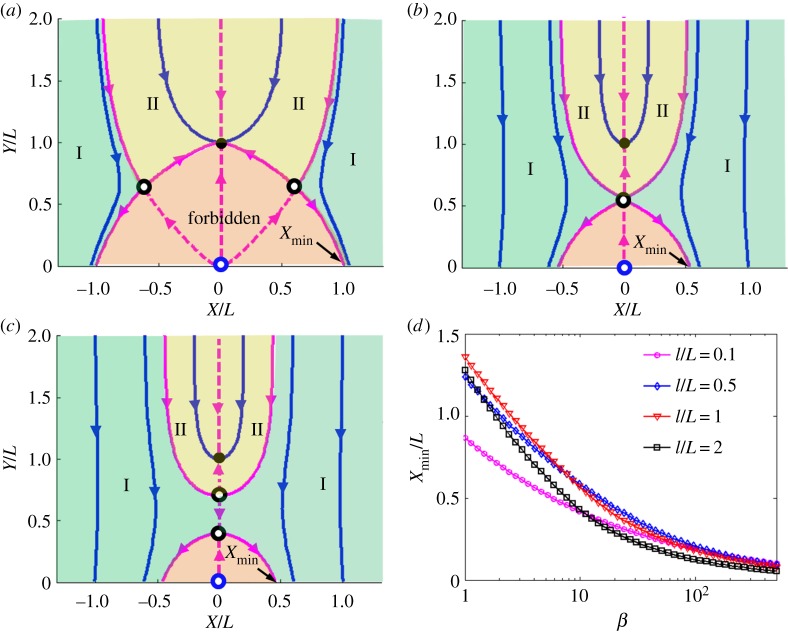
The phase portrait of dynamic system (4.1) for different parameters *β*. (*a*) *β*=2.5, (*b*) *β*=11.1 and (*c*) *β*=15.0. In *a*–*c*, the blue lines are the trajectories of the centre of mass of the incoming nanorods. The impermeable boundary is located at *Y*/*L*=−0.5 which is not shown in the graphs. The pink region is forbidden for the incoming nanorod implying that the nanorod would never land in this region. The purple lines are separatrices for this two dimensional dynamic system. The solid purple lines divide the phase portrait into two regions (I and II). If the nanorod starts its motion from region I, it will come to the boundary. The nanorod starting in region II will land on top of the pinned nanorod. The black dot (0, 1) is the energy minimum corresponding to point 1 in figure 4. This point is the attractor of this dynamic system. The empty blue circle (0, 0) is the energy maximum corresponding to point 3 in figure 4. It is an unstable stationary point for this dynamic system. The empty black circles are the saddle points and are the interceptions of the separatrices. (*d*) The plot of the minimum distance separated the incoming nanorod and the pinned nanorod as a function of *β* for the pair of nanorods with different length ratios *l*/*L*.

When the field gradient is strong and parameter *β* is much greater than 1, the energy surface deforms significantly. For example, in figure 4*c* corresponding to *β*=25, the energy surface in the vicinity of point 1 forms a funnel-like singularity. Thus, with a slim chance of success the incoming nanorod would land on top of the pinned one. The region where the field gradient governs the positioning of the incoming nanorod spreads over the larger region where the arrows in figure 4*c* are pointing almost straight down. Even for extremely high *β*, the incoming nanorod can never land right next to the pinned one owing to the inherent repulsion between them. However, the variation of parameter *β* provides a possibility to control the inter-rod distance, which will be further discussed in the next section (figure 5*d*).

This analysis of the energy landscape favours a possibility of placement of the incoming nanorod side by side next to the pinned one. In applications, it is important to control the nanorod placement, hence it is necessary to specify the range of initial positions of the incoming nanorod (*X*, *Y*) leading to its landing on the boundary or on top of the pinned nanorod.

## 5. Dynamics of magnetic nanorods: phase portrait

Assume that the nanorods are suspended in a simple Newtonian fluid with viscosity *η*. In the limit of low Reynolds numbers when the inertial force is much smaller than the viscous force, the velocity field of the incoming nanorod is described by the following dynamic system [49]:

5.1 $$\begin{array}{rl} \frac{\text{d}X}{\text{d}t} & =-\frac{1}{\gamma_{X}}\frac{\partial U(X,Y)}{\partial X},\;\gamma_{X}=\frac{4\pi \eta l}{\ln(2l/d)+0.5}\\ \text{and}\;\frac{\text{d}Y}{\text{d}t} & =-\frac{1}{\gamma_{Y}}\frac{\partial U(X,Y)}{\partial Y},\;\gamma_{Y}=\frac{2\pi \eta l}{\ln(2l/d)+0.5}, \end{array}\}$$

where *γ*_*X*_ and *γ*_*Y*_ are the translational drag coefficients of the nanorods moving in the *X* and *Y* directions, respectively [49], and *t* is the time. The dynamic system (4.1) was analysed numerically: each pair of initial conditions (*X*_0_, *Y*_0_) generated a trajectory. Two nanorods with the same length (*L*=*l*), diameter *d* and magnetization *M* were used in these calculations. The aspect ratio *l*/*d* was taken as 25. As the centre of coordinates was chosen at the centre of mass of the pinned nanorod, the substrate was located at *Y*/*L*=−1/2. Therefore, when the incoming nanorod reaches position *Y*/*L*=0, it lands on the boundary; the static friction from the boundary is expected to pin it there. The calculation will stop at this moment of time. In figure 5*a*–*c*, the blue line solutions describe the trajectories (*X*(*t*), *Y*(*t*)) of the incoming nanorods. In order to distinguish the scenarios of the nanorod landing, we have to analyse all initial conditions and classify the trajectories on the phase portrait of equation (4.1).

We first look at the *Y*-axis of the phase portrait. Along this axis, the following equality *F*_*X*_(0,*Y*)=0 holds true. This equality implies that magnetic force is always directed along the *Y*-axis and that the nanorods starting at any point (0, *Y*_0_) will move along the *Y*-axis.

There are two singular points of this system where the *Y*-component of magnetic force *F*_*Y*_ goes to infinity. The first singular point, shown as the black dot with coordinates (0, 1) in figure 5*a*–*c*, corresponds to the energy minimum. It is the attractor of the dynamic system: the pinned nanorod attracts the incoming one and forces it to land on top. The local trajectories converge towards this attractor and the arrows show the velocity vectors of the incoming nanorods.

The second singular point is the centre of coordinates attached to the centre of mass of the pinned nanorod, point (0, 0). This point corresponds to the energy maximum: two nanorods which are brought together and placed right next to each other cannot stay in equilibrium. The local trajectories emanate from this unstable stationary point.

Besides these two singular points, there are two more stationary points of the dynamic system (4.1) satisfying the following equations: *F*_*X*_=0, *F*_*Y*_=0. These stationary points are the saddle points of the dynamic system. Along some sets of local trajectories, the velocity vectors are always directed towards these stationary points and along some other sets of trajectories the velocity vectors are always directed away from them. These two stationary points are marked as the empty black circles in figure 5*a*–*c*. In figure 5*b*, these two saddle points merge at the *Y*-axis.

The separatrices, the solid purple lines, are defined as the trajectories of nanorods emanating from the saddle points. The details of their calculations are given in appendix A.

## 6. Classification of the landing scenarios for the nanorods

With the aid of this analysis of the phase portrait of dynamic system (4.1), we can classify the scenarios of the nanorod landing. The solid purple separatrices in figure 5*a*–*c* divide the phase portrait onto three regions: region I (dark green), region II (dark yellow) and the forbidden region (pink) where no nanorod can reach. The nanorods are pushed away from the forbidden region by the strong repulsion from the pinned nanorod.

The boundaries of regions I, II and the forbidden region are sensitive to the applied magnetic field gradient *α* as *β* is proportional to *α*. In the limiting case *β*=0, when the external field is uniform, the two saddle points are infinitely far from each other and region II occupies an infinitely large area. This implies that the incoming nanorod will always land on top of the pinned one. This statement is supported by the energy map shown in figure 4*a*.

By increasing the field gradient, i.e. parameter *β*, one opens the possibility of landing the incoming nanorod next to the pinned one. In the phase portrait, the two saddle points are pushed to come from infinity closer to the *Y*-axis. Therefore, the area of region II shrinks and the area of region I increases to offer the incoming nanorod a possibility to land next to the pinned one.

There is a critical field gradient when the two saddle points merge at the *Y*-axis. In figure 5*b*, this case corresponds to *β*_cr_=11.1. At this critical field gradient, only one saddle point (0, *Y*_cr_) exists and it is attached to the *y*-axis. At this point, the *x*-component of the magnetic force is zero. In other words, the force caused by the interpole interactions is balanced by the force caused by the field gradient. The critical field gradient separates two topologically different portraits: when *β*<*β*_cr_ and when the expansion of region I, the contraction of region II and the forbidden region occur mainly in the *x*-direction. In the opposite case when *β*>*β*_cr_, the two saddle points move along the *Y*-axis separating region II from the forbidden region. Region I expands mainly in the *Y*-direction.

In the limit β→∞, when the field gradient is much stronger than the field of the pinned nanorod, one saddle point moves to coincide with the energy maximum at (0, 0) and the other merges with the energy minimum at (0, 1). In this limit, the areas of region II and the forbidden region shrink to zero, implying that the incoming nanorod will always land on the boundary side by side with the pinned nanorod.

**Figure 6.**
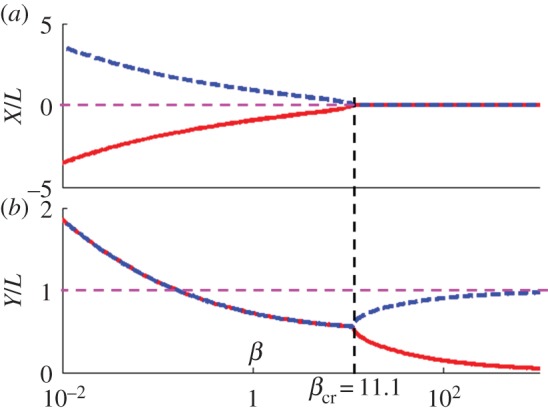
Coordinates of the two saddle points as functions of *β*. (*a*) *X*-coordinate (*b*) *Y*-coordinate. Solid red line represents saddle point 1 and dashed blue line, saddle point 2.

Figure 6 summarizes the topological change of the phase portrait. When *β*<*β*_cr_, the two saddle points are symmetric with respect to the *Y*-axis. As *β* increases, the two saddles points come closer to the *Y*-axis implying the expansion of region I and shrinkage of both region II and the forbidden region. When *β*=*β*_cr_, the two saddle points merge at the *Y*-axis and stay there as *β* further increases. When *β* goes to infinity, one saddle point moves along the *Y*-axis towards point (0, 0) and the other moves along the *Y*-axis towards point (0, 1). Simultaneously, region II and the forbidden region disappear. This topological change of the phase portrait is illustrated in the electronic supplementary material, movie S1.

For the practical applications, it is instructive to analyse the change of the half width of the forbidden region, *X*_min_, as a function of *β*. This parameter, *X*_min_, corresponds to the minimum spacing between two nanorods landed at the boundary. In figure 5*d*, we plot this minimum spacing as a function of *β* for the nanorods with different length ratios *l*/*L*. In all cases, the minimum spacing decreases as *β* increases. It follows that the minimum spacing, *X*_min_, asymptotically approaches zero as the field gradient increases. Using the relations shown in figure 5*d*, the inter-rod spacing can be controlled by varying the *β* parameter.

### 6.1 Experimental verification of different scenarios of nanorod landing

Experimental reproduction of the phase portrait requires tracking multiple pairs of incoming and pinned nanorods with the same length ratios *l*/*L* as well as with the same dimensionless parameter *β*. Therefore, figure 5*a*–*c* is difficult to reproduce experimentally. However, the trajectories of the incoming nanorods are traceable. The length ratio *l*/*L* can be measured directly from the images and only one unknown parameter *β* requires determination from the experiments. As the *β*-parameter has a strong dependence on the diameter of the pinned nanorod, *β*∝1/*d*^2^, but the dark field images do not allow one to accurately measure the nanorod diameters, we determined this parameter by fitting the experimental trajectories with the numerical solutions of equation (4.1).

**Figure 7.**
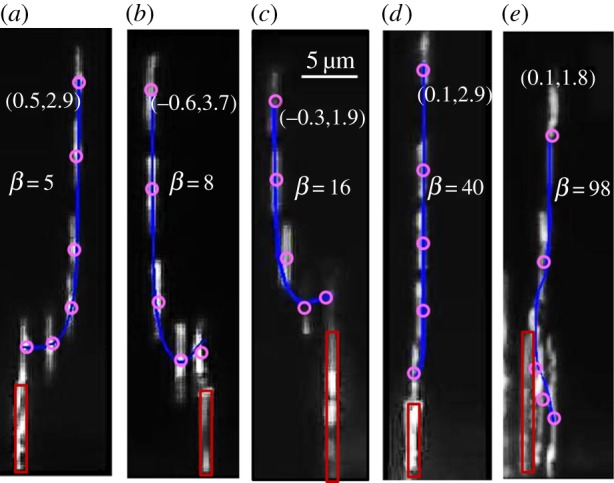
Experiments with nickel nanorods. The purple circles denote the centres of mass of the incoming nanorods. The red rectangles mark the pinned nanorods. The blue lines are the theoretical curves. The initial position of the incoming nanorod is represented by the normalized coordinates given in parentheses.

Several frames including the initial and final frames were first extracted from each video and the initial frame was then overlaid with all the following frames. Therefore, we can form an image showing the trajectory of the incoming nanorod. In figure 7, we demonstrate five composite images. These pictures illustrate different scenarios of the nanorod landing. The centre of mass of the incoming nanorod is denoted by the purple circle and the pinned nanorod is marked by the red rectangle.

Using parameter *β* as the adjustable parameter, in each case we obtained the best fits for the experimental trajectories. These best fits are shown in figure 4 as the blue lines. The theoretical curves demonstrate an excellent agreement with the experimental trajectories. Based on the proposed theory taking *β*=98, and the ratio *l*/*L*=1, the minimum spacing should be equal to *X*_min_/*L*=0.18 (figure 5*d*). The experimental inter-rod spacing extracted from figure 7*e* is equal to *X*/*L*=0.2. Thus, the theory is in excellent agreement with the experiment. This confirms the validity of the proposed model.

In figure 7*a*–*e*, the dimensionless parameter *β* increases gradually from 5 to 98. In figure 7*a*, where parameter *β* is the smallest, the field gradient is considered weak. According to the model predictions, the incoming nanorod lands directly on top of the pinned one. In figure 7*b*, parameter *β* is greater than in figure 7*a* and the incoming nanorod also starts movement from a more distant position. As a result, the trajectory of the incoming nanorod is distinct: the incoming nanorod first passes the pinned one and is tempted to land next to it. But when its head almost passes the pole of the pinned nanorod where the local field is stronger than the external one, its *Y*-component of velocity reverses the sign reflecting the change in the force field. The incoming nanorod first moves downward then drifts upward and finally jumps on top of the pinned one. These manoeuvres are illustrated by the last three purple points in figure 7*b*. A similar behaviour of a nanorod is shown in figure 7*c* with its initial position closer to the pinned one and a stronger field gradient acting on it.

**Figure 8.**
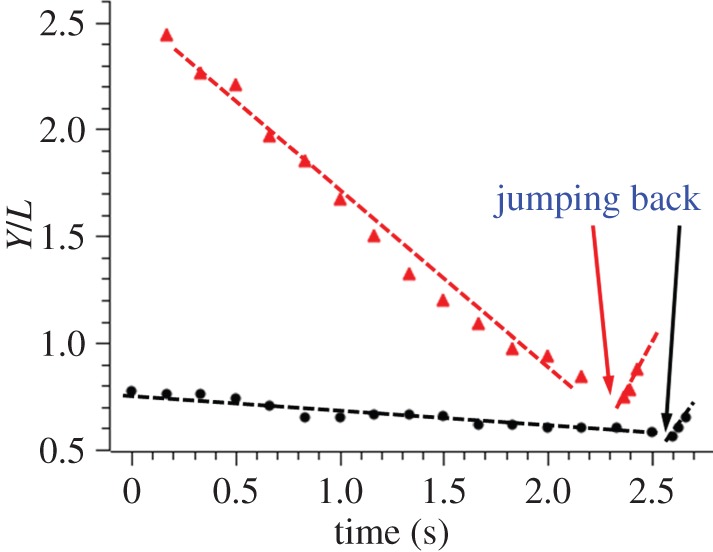
*Y*-coordinates of the incoming nanorods as a function of time for experiments. Black circles denote *β*=16 and red triangles, *β*=8 as shown in figure 7*b* and *c*, respectively.

Figure 8 shows the *Y*-coordinate of the centre of mass of the incoming nanorod as a function of time for the manoeuvres illustrated in figure 7*b,c*. The transition region, pointed to by the arrows, where the nanorod is about to change the direction of movement cannot be resolved owing to an insufficient frame rate of the camera. However, one can clearly see that the slopes, i.e. the *Y*-components of velocity, remain almost constant before and after the moment when the nanorod stops. The time needed for a nanorod to change the direction of movement is approximately 0.1 s, and after this transition period the nanorod takes on a constant velocity and quickly approaches the pinned one.

This overshooting is not related to inertia because the Reynolds number is very small [49–55]. This change of velocity direction is completely governed by the non-uniform magnetic field and can be understood by considering the interactions between the head of the incoming nanorod (−*Q*) and the tail of the pinned one (+*Q*). One can neglect the interactions between other distant poles. Following this assumption, the *Y*-component of the magnetic force between these two poles can be calculated using equations (3.1) and (3.3) as

6.1 $$F_{Y}=-\frac{\partial U(X,Y)}{\partial Y}=-\frac{\mu_{0}Q^{2}}{4\pi }\frac{Y-l/2-L/2}{{\left[X^{2}+{\left(Y-l/2-L/2\right)}^{2}\right]}^{3/2}}-Ql\alpha ,$$

where *α* is always positive. When the head of the incoming nanorod is above the tail of the pinned one and the inequality *Y* −*l*/2−*L*/2>0 holds true, the force *F*_*Y*_ is always negative indicating that the nanorod moves always downwards. If the incoming nanorod passes the pinned nanorod by an infinitesimally small distance as shown in figure 7*b,c*, the first term on the right hand side of equation (5.1) becomes positive. As the incoming nanorod keeps moving downwards, the force *F*_*Y*_ may decrease to zero and then flip the direction at some critical point. After this moment, the incoming nanorod will drift upwards and finally will jump on top of the pinned one.

The nanorods in figure 7*d,e* had the same initial *X*-coordinate (*X*/*L*=0.1). However, as the field gradient for the case in figure 7*d* is smaller relative to that in figure 7*e*, the smaller gradient was not able to defeat the force field of the pinned nanorod, and the incoming nanorod landed on top of the pinned one. By contrast, at the dimensionless field gradient *β*=98, we were able to place the nanorods side by side next to each other. Thus, the proposed theory completely explains the experimental observations and can guide the remote controlled placement of the nanorods [35].

## 7. Conclusion

We present a complete analysis of the dynamics of interacting magnetic nanorods subject to a non-uniform external magnetic field. The magnetostatic interactions between long magnetic nanorods are described through the interactions of their poles carrying ‘magnetic charges’. The energy landscape was studied theoretically and all minima and maxima of the energy surface were specified. Then we described the two-dimensional dynamics of magnetic nanorods when their resistance is controlled by the viscous drag. Using the phase portrait of this dynamic system we described the regions of initial positions from which the free nanorod can be placed sidewise next to the pinned one. This theory was then confirmed in experiments with nickel nanorods. We showed that a strong repulsion between nanorods can be defeated by applying a non-uniform magnetic field with a strong gradient in the direction of nanorod alignment. With the aid of the proposed theory, one can build different lattices, microstructures and composite materials with the controlled inter-rod spacing. Therefore, various properties of nanocomposites, such as magnetic, optical, electrical and mechanical properties, can be tuned by changing the inter-rod spacing by applying a non-uniform magnetic field during the composite formation.

## Supplementary Material

Dynamics of nanorod landing. Each trajectory in Fig 7 was generated using a movie with the same frame number

## Figures and Tables

**Figure 9.**
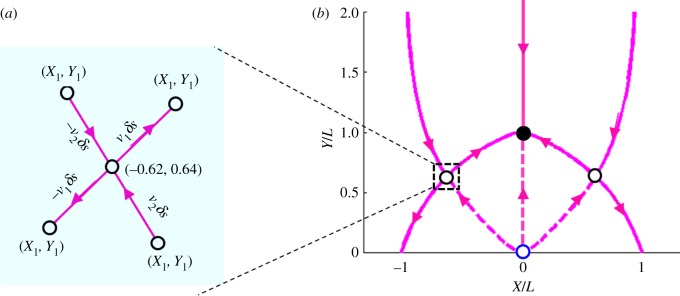
(*a*) Four movements from a saddle point. (*b*) Resulting separatrices generated using the described movement from two saddle points.

## Data accessibility

All movies used in the presented analysis are available in the electronic supplementary material.

## Appendix A. Numerical generation of separatrices

To generate the separatrices numerically, we have to linearize the dynamic system in the vicinities of the saddle points [51]. This linearization results in the following equations

A 1 $${\left(\begin{array}{c} \frac{\gamma_{X}\text{d}X}{\text{d}t}\\ \frac{\gamma_{Y}\text{d}Y}{\text{d}t} \end{array}\right)=\left(\begin{array}{cc} \frac{\partial F_{X}}{\partial X} & \frac{\partial F_{X}}{\partial Y}\\ \frac{\partial F_{Y}}{\partial X} & \frac{\partial F_{Y}}{\partial Y} \end{array}\right)|}_{\left(X^{*},Y^{*}\right)}\cdot \left(\begin{array}{c} X\\ Y \end{array}\right),$$

where all partial derivatives in the matrix are evaluated at the saddle points (*X**, *Y**). Each saddle point generates four branches of separatrices shown in figure 5*a*–*c*. Whether the velocity vector is pointing towards or away from the saddle point depends on the sign of the corresponding eigenvalue with the ‘plus’ implying the divergence of the vector field and ‘minus’ implying convergence of the vector field towards the saddle point. After this movement from the singular points, the separatrices can be generated by solving equation (4.1) with the initial condition at (*X*_1_, *Y*_1_).

In order to specify the trajectories, we calculate two eigenvectors ***v***_1,2_ of matrix (A 1). Near the saddle points, each branch of the separatrix is approximated by a straight line specified by an eigenvector. As a result, the movement of the nanorod from the saddle point (*X**, *Y**) is defined as:

A 2 $$(X_{1},Y_{1})=(X^{*},Y^{*})\pm (\nu_{1,2})\delta s,$$

where *δs*=(*δX*^2^+*δY* ^2^)^1/2^(*δX*=*X*_1_−*X**, *δY* =*Y*_1_−*Y**) is the small displacement from saddle point (*X**, *Y**) to point (*X*_1_, *Y*_1_). In all the calculations, we used *δs*=5×10^−3^. There are four different shifting vectors: ***v***_1_*δs*,−***v***_1_*δs*,***v***_2_*δs*,−***v***_2_*δs* specifying the movement (*X*_1_, *Y*_1_).

Worked example: taking the case *β*=2.5 as an example, two saddle points are (±0.62, 0.64).

The linearized matrix at these two points is calculated as

A 3 $${\left(\begin{array}{cc} \frac{(1/\gamma_{X})\partial F_{X}}{\partial X} & \frac{(1/\gamma_{X})\partial F_{X}}{\partial Y}\\ \frac{(1/\gamma_{Y})\partial F_{Y}}{\partial X} & \frac{(1/\gamma_{Y})\partial F_{Y}}{\partial Y} \end{array})\right|}_{\left(\pm 0.62,0.64\right)}=\left(\begin{array}{cc} 0.0068 & \mp 0.0283\\ \mp 0.0438 & -0.0105 \end{array}\right).$$

The eigenvalues and eigenvectors of this matrix are given as

A 4 $$\lambda_{1}=0.0344,\;\nu_{1}=\left(\begin{array}{c} 0.7158\\ \mp 0.6983 \end{array}\right)\text{and}\;\lambda_{2}=-0.0381,\;\nu_{2}=\left(\begin{array}{c} \pm 0.5335\\ 0.8458 \end{array}\right).$$

Near the saddle points, each branch of the separatrix is approximated by a straight line following the direction of the eigenvector. As a result, the movement (A 2) of the nanorod from the saddle points (−0.62, 0.64) is given as

A 5 $$\left(\begin{array}{c} X_{1}\\ Y_{1} \end{array}\right)=\left\{ \begin{array}{c} \left(\begin{array}{c} X^{*}\\ Y^{*} \end{array}\right)\pm \nu_{1}\delta s=\left(\begin{array}{c} -0.62\\ 0.64 \end{array}\right)\pm \left(\begin{array}{c} 0.7158\\ 0.6983 \end{array}\right)\delta s\;(\lambda_{1}>0,\;\text{pointing outwards})\\ \left(\begin{array}{c} X^{*}\\ Y^{*} \end{array}\right)\pm \nu_{2}\delta s=\left(\begin{array}{c} -0.62\\ 0.64 \end{array}\right)\pm \left(\begin{array}{c} -0.5335\\ 0.8458 \end{array}\right)\delta s\;(\lambda_{2}>0,\;\text{pointing inwards}). \end{array}\right.$$

Taking *δs*=5×10^−3^, this movement is illustrated in figure 9*a*.

With the four points obtained, one can generate four branches of separatrices by taking points (*X*_1_, *Y*_1_) as the initial positions for the dynamic system. The results are shown in figure 9*b*. Four other branches of separatrices can be generated at saddle point (0.62, 0.64) using the same type of movement. Besides those generated separatrices, the *Y*-axis is a natural separatrix as discussed in the main text.
