# Supplementary material for: A gradient field defeats the inherent repulsion between magnetic nanorods
Source: R Soc Open Sci. 2014 Oct 8;1(2):140271. doi: 10.1098/rsos.140271 (PMC4448895; doi:10.1098/rsos.140271)
Supplement: Dynamics of nanorod landing. Each trajectory in Fig 7 was generated using a movie with the same frame number [file rsos140271supp1.docx]

# A gradient field defeats the inherent repulsion between magnetic nanorods

**Supporting movie**

Yu Gu, Ruslan Burtovyy, John Custer, Igor Luzinov and Konstantin G. Kornev

Department of Materials Science and Engineering

Clemson University, SC, 29634

In this model, two nanorods have the same length, diameter and magnetization. In the movie, the black line corresponds to the contour *F_Y_*=0, the red lines correspond to the contours *F_X_*=0. Purple lines are the separatrices as introduced in the main text. The separatrices in the forbidden region are not shown in the movie. The blue lines are the trajectories of the incoming nanorods. The dimensionless factor *β* is varied.

The video illustrates the topological change of the phase portrait as parameter *β* increases. The two saddle points move along the contours *F_X_*=0 as the contour *F_Y_*=0 deforms. Initially, region I (from which the incoming nanorod lands on top of the pinned one) and forbidden region occupy most of the area. As *β* increases, region I and the forbidden regions keep shrinking while region II (from which the incoming nanorod lands on the boundary) keeps expanding.

A series of movies corresponding to Fig.7 a)-e) with the tag indicating the chosen scenario Fig. 7a) – e) was used to obtain the nanorod trajectories.
